# Investigating *PRDM8* DNA Methylation in Peripheral Tissues in Borderline Personality Disorder: Association with Symptom Severity but Not Adverse Childhood Experiences

**DOI:** 10.3390/brainsci15090950

**Published:** 2025-08-30

**Authors:** Annika Bender, Laila Bertele, Mirac Nur Musaoglu, Sarah Pasche, Susanne Edelmann, Vanessa Nieratschker

**Affiliations:** 1 Department of Psychiatry and Psychotherapy, Eberhard Karls University of Tuebingen, 72076 Tuebingen, Germanymirac.nur.musaoglu@med.uni-tuebingen.de (M.N.M.);; 2 German Center for Mental Health, Partner Site Tuebingen, 72076 Tuebingen, Germany

**Keywords:** borderline personality disorder, adverse childhood experiences, dialectic behavioral therapy, DNA methylation, *PRDM8*

## Abstract

Background: Borderline Personality Disorder (BPD) is a complex psychiatric condition with multifactorial origins, with a high proportion of patients reporting early trauma. Stressors such as adverse childhood experiences (ACEs) can shape the epigenetic landscape including DNA methylation (DNAm) and act on gene expression. DNAm is increasingly being investigated as a molecular link between environmental exposures such as ACE and psychiatric outcomes. Differential DNAm of the gene PR domain zinc finger protein 8 (*PRDM8*), a histone methyltransferase, has recently been reported to be sensitive to early life trauma. Its role in BPD, especially in the context of ACE, remains to be elucidated. Methods: This study investigated DNAm patterns of *PRDM8* in peripheral blood and saliva obtained from BPD patients undergoing Dialectic Behavioral Therapy (DBT) compared to healthy control (HC) participants. Associations with ACE and BPD symptom severity were assessed, and therapy-related changes in DNAm were examined. Results: At baseline, BPD patients demonstrated significant hypomethylation of *PRDM8* in blood relative to the HC group. Following DBT, a nominally significant increase in DNAm was observed, aligning with inversely correlated symptom severity. No significant differences in saliva were detected. ACE was not associated with *PRDM8* DNAm. Conclusions: Our findings suggest that *PRDM8* DNAm might be associated with BPD and therapeutic intervention but not with ACE. Together with prior research, the results underscore the importance of future investigation of gene–environment interactions and the functional significance of *PRDM8* regulation in the pathophysiology of BPD.

## 1. Introduction

Borderline Personality Disorder (BPD) is a severe and complex mental disorder characterized by instability in affect regulation, impulse control, interpersonal relationships, and self-image [1]. BPD affects approximately 0.7–5.8% of the general population, with higher rates of up to 10% in psychiatric outpatients and 20% of inpatients [2,3,4,5]. The etiology and pathophysiology of BPD is multifactorial, including genetic vulnerability, structural and functional brain alterations, and environmental stressors, as well as accompanying epigenetic modifications that may alter gene expression (for reviews see, e.g., [6,7,8]). In recent years, epigenetic mechanisms have received increasing attention for their role in mediating the long-term impact of environmental exposures on psychological and neurobiological development [9]. Among these mechanisms, DNA methylation (DNAm) has been particularly well studied. Multiple genes have shown differential DNAm in individuals with BPD, including genes associated with estrogen regulation, neurotransmitter systems, neurogenesis, immune response and cell differentiation [10,11,12,13,14,15,16].

DNAm influences gene expression and is known to provide a biological record of environmental influences, such as adverse childhood experiences (ACEs; Refs. [17,18,19]). ACE, including emotional and physical abuse or neglect and sexual abuse, is reported by 30–90% of individuals with BPD, depending on trauma type [3]. In the context of BPD, various pathophysiological changes on the neurobiological, neuroanatomical, and epigenetic levels have been reported to depend on or interact with ACE [20]. Thus, such early environmental stressors have been implicated as significant risk factors in the development of BPD [21,22]. In this context, DNAm is increasingly being investigated as a potential mechanism linking ACE to later psychiatric outcomes [23,24,25,26,27].

One gene of interest is PR domain zinc finger protein 8 (*PRDM8*), where DNAm changes have been implicated as a molecular signature of early life trauma. A recent study [28] found that *PRDM8* DNAm (cg18954401) was associated with early trauma exposure related to genocide. Previously, elevated *PRDM8* DNAm (cg05059566) in both trauma-exposed mothers and their offspring has been reported [29], highlighting the gene’s potential relevance in this context. *PRDM8* encodes a histone methyltransferase that primarily represses transcription by methylation of lysine 9 of histone H3 (H3K9) [30,31]. Increased *PRDM8* gene expression has been linked to elevated H3K9 methylation levels in humans and has been implicated in neuronal development, differentiation, and neurogenesis across multiple mouse studies [30,31,32,33,34,35]. Moreover, in humans, *PRDM8* has been associated with immune signaling pathways [36] and neurodegenerative disorders [37,38]. Given its roles in neurodevelopment and its sensitivity to early trauma, *PRDM8* is a compelling candidate for investigating the molecular underpinnings of BPD.

Dialectic behavioral therapy (DBT), a structured, evidence-based psychotherapy for BPD developed by M. Linehan specifically for BPD patients exhibiting suicidality and self-harm, shows strong empirical evidence of generally good efficacy [39,40,41,42]. For inpatient settings, DBT has been modified as a 12-week intervention, involving emotion regulation and mindfulness training [43]. Notably, epigenetic mechanisms have also been linked to therapeutic response, with several studies reporting associations between DNAm changes and DBT outcomes [14,15,44,45], further underscoring the potential relevance of DNAm in BPD pathophysiology.

Building upon the association of *PRDM8* DNAm with ACE, a major risk factor of later BPD development, the present study examines DNAm patterns of *PRDM8* in individuals diagnosed with BPD compared to healthy control (HC) individuals, considering their history of ACE. Additionally, potential therapy-associated changes in *PRDM8* DNAm are investigated within the BPD group and through comparison of post-treatment levels with those of HC participants. Overall, this study aims to explore whether *PRDM8* DNAm may serve as a molecular link between ACE exposure, BPD diagnosis, and therapy effects.

## 2. Materials and Methods

### 2.1. Study Cohort

The cohort (partially) overlaps with previous studies [14,16,24], including 93 European participants (*n* = 40 BPD patients, *n* = 53 HC participants) being recruited between 2013 and 2016 as previously described [14,15]. Out of the 40 BPD patients who were hospitalized for a 12-week DBT program, only 24 completed the therapy and were thus available for pre (T1)-/post (T2)-therapy analyses. HC participants were assessed only at baseline (T1). BPD diagnosis was determined by the International Personality Disorder Examination (IPDE; Ref. [46]) according to the criteria of the fourth edition of the Diagnostic and Statistical Manual of Mental Disorders (DSM-IV; Ref. [1]. The age and sex of the HC participants, who had no history of psychiatric disorders, were matched to those of the BPD patients. Written informed consent was provided by each person who took part in the study. The study was approved by the University of Tuebingen’s local ethics committee and performed in accordance with the Declaration of Helsinki.

### 2.2. Measures

#### 2.2.1. Questionnaires

The Childhood Trauma Questionnaire (CTQ), which has five subscales (emotional, physical, and sexual abuse, as well as emotional and physical neglect), was used to measure ACE [47,48]. Participants were considered to have high levels of ACE if they reached a moderate score on at least one of the subscales (sexual abuse: >8; physical abuse: >10; physical neglect: >10; emotional abuse: >13; emotional neglect: >15; Refs. [47,48]). To assess symptom severity, the Borderline Symptom List (BSL-23; Ref. [49]) was used.

#### 2.2.2. PRDM8 DNAm Analysis in Saliva and Whole Blood

Ethylenediaminetetraacetic (EDTA) tubes (SARSTEDT AG & Co. KG, Nümbrecht, Germany) were used to collect peripheral venous blood samples in the first (T1) and last week of therapy (T2), which were kept at −80 °C until further use. Following the manufacturer’s instructions, genomic DNA was extracted from the samples using the QIAmp DNA Blood maxi Kit (Qiagen, Hilden, Germany; Refs. [50,51]). The OraGene^®^ DNA saliva kit (DNA Genotek, Ottawa, ON, Canada) was used to collect saliva samples, which were then stored at −80 °C until further use. The prepIT L2P solution (DNA Genotek) was used to isolate genomic DNA in accordance with the manufacturer’s protocol. A Qubit 4.0 Fluorometer (Life Technologies, Carlsbad, CA, USA) was used to measure the yield and purity of DNA. 500 ng of genomic DNA were bisulfite converted using the EpiTect Fast Bisulfite Kit (Qiagen) and stored at −20 °C until further use [52,53].

A region on the minus strand of chromosome 4q21.21 (GRCh38/hg38: chr4: 802,034,67–802,037,80), located in the last exon of the gene *PRDM8* was amplified (forward primer: 5′-TTTTTGTGTGTGAGTGTGTTT-3′, reverse primer: 5′-Biotin-CCCATCTACAATAAATCCTTC 3′ (Metabion, Planegg/Steinkirchen, Germany)), spanning the CpG sites cg18954401 (chr4: 80,203,613, further referred to as CpG site 1), and two unannotated CpG sites (CpG site 2: chr4: 80,203,600 and CpG site 3: chr4: 80,203,598) by performing region-specific PCRs using the PyroMark PCR Kit (Qiagen) according to the manufacturer’s protocol. The success and specificity of each PCR were verified by gel electrophoresis. Using the PyroMark Q24 system and software (Version 2.0.7, Qiagen), DNAm levels of the corresponding CpG sites of interest were analyzed by pyrosequencing in technical duplicates that differed by no more than 3% (sequencing primer: 5′-GGGGTGTAAGGAA-3′ (Metabion)). A titration assay was performed using standardized bisulfite converted control DNA samples (EpiTect Control DNA, Qiagen; Ref. [54]) in order to validate the custom pyrosequencing assay. To indicate missing values, sample sizes per group and tissue are visualized in Tables S3 (diagnosis-based comparisons) and S5 (ACE-based comparisons).

### 2.3. Data Analyses

#### 2.3.1. Demographic and Clinical Data

Normality of all variables was tested using the Shapiro–Wilk test. As most variables deviated from normality, all variables were treated as non-normal for downstream analyses to ensure comparability of test results. Differences in the sociodemographic and questionnaire data of BPD and HC participants were thus assessed using the Wilcoxon rank sum test or, for factorial variables, Fisher’s exact test.

Since only 10 HC participants had high levels of ACE, and only 2 BPD participants showed low levels of ACE (Table 1), a comparison of BPD and ACE was not possible to perform simultaneously. Thus, the effects of diagnosis and ACE status had to be analyzed in separate group comparisons.

#### 2.3.2. Statistical Analyses

All analyses were conducted using the software environment R (version 4.4.1). For all statistical analyses, the significance level α was set to 0.05. A DNAm mean of each respective CpG site was obtained by averaging the two technical replicates of the measured DNAm per individual and CpG site for each tissue, respectively. These per-site DNAm values were then averaged across all examined CpG sites to derive a mean DNAm level per individual and tissue, hereinafter referred to as the *PRDM8* blood and saliva DNAm level. Prior to averaging, correlations between CpG sites were assessed using Spearman’s rank correlation test (Table S2). Following Bonferroni correction, in the BPD-T1 group, no significant correlations were observed between CpG sites 1 and 3 in blood, and in the BPD-T2 group, no significant correlations were found for CpG sites 1 and 3, or for CpG sites 2 and 3 in blood. Consequently, the analyses including these variables were also conducted on the level of individual CpG sites to determine whether findings based on *PRDM8* DNAm values aligned with site-specific patterns. Therefore, correction for multiple testing according to the Benjamini–Hochberg procedure [55] was performed within each analysis block. Analysis blocks (i.e., hypothesis families) were defined as follows: (1) a cross-sectional analysis comparing HC and BPD-T1 participants using the Wilcoxon rank sum test with continuity correction; (2) a longitudinal within-subject analysis comparing BPD-T1 and BPD-T2 participants using the Wilcoxon signed rank exact test; and (3) a comparison of HC and BPD-T2 participants using the Wilcoxon rank sum test with continuity correction. In addition to splitting the cohort according to diagnosis and time point, the cohort was split according to ACE status, irrespective of diagnosis, for comparison of *PRDM8* DNAm with respect to ACE for the cross-sectional analysis at baseline. Lastly, possible associations of CTQ and BSL-23 scores with *PRDM8* DNAm at T1 were explored by Spearman’s rank correlation test with continuity correction.

## 3. Results

### 3.1. Demographic and Clinical Information

The cohort characteristics with respect to the HC and BPD groups are visualized in Table 1.

The HC group showed a median age (± interquartile range, IQR) of 26.00 = 9.00 years and the BPD group of 28.00 ± 10.25 years, reflecting no significant age differences (W = 1257, *p* = 0.1266). Neither did the groups differ in their sex distribution (Fisher’s exact test *p* = 0.5751) as 46 out of the 53 HC individuals and 33 out of the 40 BPD patients were female. BPD participants were significantly more likely to exhibit high levels of ACE (Fisher’s exact test *p* < 0.0001) with 38 BPD patients and only 10 HC participants reaching the defined threshold of at least a moderate score in at least one of the CTQ subscales. Accordingly, the BPD group reported a significantly (W = 1996.50, *p* < 0.0001) higher median CTQ score (± IQR) of 59.00 (± 25.25) compared to the HC group’s median score of 32.00 (± 9.00). Lastly, the BPD group scored significantly higher in all CTQ subscale scores (emotional abuse: W = 1898.00, *p* < 0.0001, emotional neglect: W = 1830.00, *p* < 0.0001, physical abuse: W = 1713.50, *p* < 0.0001, physical neglect: W = 1717.50, *p* < 0.0001, sexual abuse: W = 1658.50, *p* < 0.0001, for medians ± IQR see Table 1).

### 3.2. Severity of Borderline Symptomatology

The median BSL-23 scores (± IQR) of all groups are displayed in Table S3. BPD patients showed significantly higher levels of BPD symptomatology at baseline compared to HC individuals (*n*_HC_ = 39, *n*_BPD_ = 39, W *=* 1520.00, *p* < 0.0001; median BSL-23 score *±* IQR; HC: 0.09 *±* 0.18, BPD-T1: 2.57 *±* 1.02). Over the course of DBT, BPD patients’ symptom severity improved significantly (*n*_BPDpairs_ = 23, V *=* 268.00, *p =* 0.0194; median BSL-23 score *±* IQR; BPD-T1: 2.57 *±* 1.02, BPD-T2: 1.78 *±* 1.04). However, it remained significantly elevated compared to the HC group’s baseline (*n*_HC_ = 39, *n*_BPD-T2_ = 23, W *=* 14.00, *p* < 0.0001; median BSL-23 score *±* IQR; HC: 0.09 *±* 0.18, BPD-T2: 1.78 *±* 1.04).

### 3.3. DNAm Levels of PRDM8 in BPD Patients and HC Individuals

**Figure 1:**
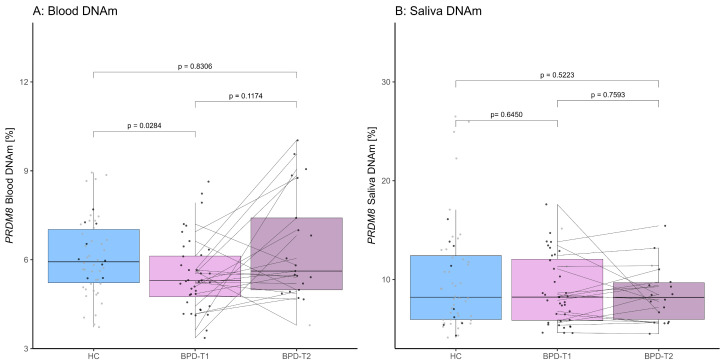
Boxplots depicting *PRDM8* DNAm across the HC, BPD-T1 and BPD-T2 groups (for median DNAm levels [%] ± IQR refer to Table S2). Data points are colored by ACE status: black = high ACE, grey = low ACE. Lines connect data points from the same individuals across T1 and T2. (**A**) *PRDM8* DNAm levels in blood (*p*-value adjusted for multiple testing). (**B**) *PRDM8* DNAm levels in saliva.

The group’s median *PRDM8* DNAm levels (± IQR) of both tissues are depicted in Figure 1 and in Table S3. *PRDM8* blood DNAm analysis revealed a significant hypomethylation in BPD patients at baseline as compared to the HC individuals (*n*_HC_ = 50, *n*_BPD_ = 40, W *=* 697.50, *p =* 0.0142, *p*_adj_ = 0.0284; median DNAm level [%] ± IQR blood: HC: 5.93 ± 1.81, BPD-T1: 5.29 ± 1.37). The same results were obtained when performing this analysis for each CpG site, respectively (CpG site 1: W *=* 757.00, *p =* 0.0489, *p*_adj_ = 0.0489; CpG site 2: W *=* 753.00, *p =* 0.0453, *p*_adj_ = 0.0489; CpG site 3: W *=* 690.50, *p =* 0.0121, *p*_adj_ = 0.0284; for CpG site-specific median DNAm level refer to Table S3). In saliva *PRDM8* DNAm, on the other hand, we did not observe any cross-sectional differences (*n*_HC_ = 52, *n*_BPD_ = 40, W *=* 981.00, *p =* 0.6450; median DNAm level [%] ± IQR saliva: HC: 8.21 ± 6.47, BPD-T1: 8.22 ± 6.19).

BPD patients showed a statistical trend of *PRDM8* DNAm increase over DBT in their blood, which, however, did not survive correction for multiple testing (*n*_BPDpairs_ = 21, V = 68.00, *p* = 0.0587, *p*_adj_ = 0.1174; median DNAm level [%] ± IQR blood: BPD-T1: 5.29 ± 1.37, BPD-T2: 5.62 ± 2.43). This effect was driven by a significant difference in CpG site 1, which also did not survive correction for multiple testing, whereas DNAm of CpG site 2 and CpG site 3 showed unchanging levels over the course of therapy (CpG site 1: V = 60.00, *p* = 0.0301, *p*_adj_ = 0.1174; CpG site 2: V = 92.00, *p* = 0.2756, *p*_adj_ = 0.3675; CpG site 3: V = 120.00, *p* = 0.8486, *p*_adj_ = 0.8486; for CpG site-specific median DNAm level [%] ± IQR refer to Table S3). *PRDM8* DNAm levels in saliva remained stable over time (*n*_BPDpairs_ = 18, V = 125.00, *p* = 0.7593; median DNAm level [%] ± IQR saliva: BPD-T1: 8.22 ± 6.19, BPD-T2: 8.91 ± 3.71).

*PRDM8* DNAm levels between HC and BPD-T2 participants displayed no significant difference (*n*_HC_ = 52, *n*_BPD-T2_ = 21, W = 568.00, *p* = 0.8306, *p*_adj_ = 0.8306; median DNAm level [%] ± IQR blood: HC: 5.9 ± 1.81, BPD-T2: 5.62 ± 2.43). No differences emerged when performing this analysis in a CpG-wise manner, except for a trend of differential DNAm in CpG site 3, which did not survive correction for multiple testing (CpG site 1: W = 531.00, *p* = 0.8211, *p*_adj_ = 0.8306; CpG site 2: W = 628.50, *p* = 0.3403, *p*_adj_ = 0.6806; CpG site 3: W = 704.00, *p* = 0.0606, *p*_adj_ = 0.2423; for CpG site-specific median DNAm level [%] ± IQR refer to Table S3). In saliva, *PRDM8* DNAm levels again did not indicate differences between the HC and BPD-T2 groups (*n*_HC_ = 52, *n*_BPD-T2_ = 18, W = 599.00, *p* = 0.5223; median DNAm level [%] ± IQR saliva: HC: 8.21 ± 6.47, BPD-T2: 8.91 ± 3.71).

**Figure 2:**
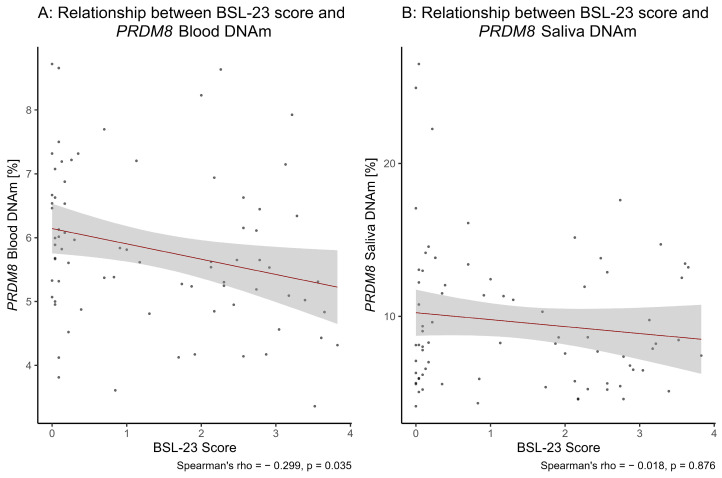
(**A**) The significant inverse correlation of the BSL-23 score and *PRDM8* blood DNAm pre-therapy is displayed (Spearman’s rank correlation test, *p*-value adjusted for multiple testing). (**B**) No association of the BSL-23 score and *PRDM8* saliva DNAm was found pre-therapy (Spearman’s rank correlation test).

Lastly, we observed a significant correlation of the BSL-23 score with *PRDM8* blood DNAm at baseline (S = 95008.00, Spearman’s rho = −0.30, *p* = 0.0087, *p*_adj_ = 0.0350; Figure 2). CpG-wise analyses revealed a statistical trend of correlation for CpG site 1, which did not survive correction for multiple testing and otherwise no significant associations (CpG site 1: S = 88578.00, Spearman’s rho = −0.21, *p* = 0.0674, *p*_adj_ = 0.1080; CpG site 2: S = 80759.00, Spearman’s rho = −0.10, *p* = 0.3712, *p*_adj_ = 0.3712; CpG site 3: S = 87884.00, Spearman’s rho = −0.20, *p* = 0.0810, *p*_adj_ = 0.1080; for CpG site-specific median DNAm level [%] ± IQR refer to Table S3). In saliva, we detected no correlation of the BSL-23 score with *PRDM8* DNAm levels at baseline (S = 77453.00, Spearman’s rho = −0.02, *p* = 0.8758; Figure 2).

### 3.4. PRDM8 DNAm Levels in the Context of ACE

The group characteristics emerging after reclassifying the cohort according to low vs. high levels of ACE are shown in Table S4. No significant difference in sex between the individuals with low (41 out of 45 female) and high levels of ACE (38 out of 48 female) was confirmed by Fisher’s exact test (*p* = 0.1487). However, the group with high levels of ACE was significantly older (median ± IQR = 28.50 ± 13.23 years) than the group with low levels of ACE (median ± IQR = 25.00 ± 9.00 years; W = 693.50, *p* = 0.0029). As expected, the groups differed significantly in the proportion of BPD patients (Fisher’s exact test *p* < 0.0001), with the group exhibiting high levels of ACE containing 38 BPD patients out of 48 participants, whereas the group with low levels of ACE included 2 BPD patients out of 45 participants. In the group with high levels of ACE, we observed a significantly higher CTQ total score (median ± IQR = 57.00 ± 27.50) as compared to the group with low levels of ACE (median ± IQR = 30.00 ± 7.00; W = 48.50, *p* < 0.0001). Accordingly, the groups differed significantly in all CTQ subscale scores (emotional abuse: W = 150.50, *p* < 0.0001, emotional neglect: W = 194.50, *p* < 0.0001, physical abuse: W = 402.50, *p* < 0.0001, physical neglect: W = 425.00, *p* < 0.0001, sexual abuse: W = 516.00, *p* < 0.0001, for respective median ± IQR see Table S4). The ACE-based group’s median *PRDM8* DNAm levels (± IQR) of both tissues assessed, and the median BSL-23 scores (± IQR) are displayed in Table S5.

*PRDM8* DNAm levels in blood were comparable in participants with low vs. high levels of ACE (*n*_lowACE_ = 43, *n*_highACE_ = 47, W = 1172.50, *p* = 0.1920, *p*_adj_ = 0.3441; median DNAm level [%] ± IQR blood: low ACE: 5.7 ± 1.65, high ACE: 5.38 ± 1.55). This result was reflected in the respective CpG-wise analyses (CpG site 1: W = 1151.00, *p* = 0.2581, *p*_adj_ = 0.3441; CpG site 2: W = 1151.00, *p* = 0.2581, *p*_adj_ = 0.3441; CpG site 3: W = 1117.00, *p* = 0.3919, *p*_adj_ = 0.3919; for CpG site-specific median DNAm levels [%] ± IQR refer to Table S5) and in *PRDM8* saliva DNAm levels (*n*_lowACE_ = 45, *n*_highACE_ = 47, W = 1193.00, *p* = 0.2932; median DNAm level [%] ± IQR saliva: low ACE: 9.03 ± 5.92, high ACE: 7.88 ± 6.50).

In line with these findings, the CTQ score was neither significantly associated with *PRDM8* blood DNAm levels (S = 136,623.00, Spearman’s rho = −0.12, *p* = 0.2419; Figure S1), nor with any of the respective CpG sites alone (CpG site 1: S = 140,683.00, Spearman’s rho = −0.16, *p* = 0.1368, *p*_adj_ = 0.4838; CpG site 2: S = 128,901.00, Spearman’s rho = −0.06, *p* = 0.5676, *p*_adj_ = 0.5676; CpG site 3: S = 131,016.00, Spearman’s rho = −0.08, *p* = 0.4623, *p*_adj_ = 0.5676). Moreover, we did not find any association of CTQ score and *PRDM8* DNAm levels in saliva (S = 136,995.00, Spearman’s rho = −0.06, *p* = 0.5979; Figure S1).

## 4. Discussion

This study aimed to investigate *PRDM8* DNAm patterns in BPD patients undergoing DBT in comparison to HC participants, and to examine the association of DNAm levels with exposure to ACE. At baseline, BPD patients exhibited significant hypomethylation of *PRDM8* in whole blood compared to the HC group across all CpG sites assessed. A within-subject analysis revealed a strong trend toward increased *PRDM8* DNAm levels following treatment, driven by a significant difference at CpG site 1, although this increase in DNAm post-treatment did not remain significant after correction for multiple testing. No significant difference was detected when comparing post-treatment BPD patients’ *PRDM8* DNAm levels to baseline levels of the HC group, although a subtle trend towards differential DNAm at CpG site 3 was noted prior to correction for multiple testing.

Diagnosis-related *PRDM8* DNAm differences were observed in blood, whereas in saliva, no differences were found between the diagnostic groups in any of the conducted analyses. Although correlations between CpG sites were consistently high across all groups in saliva, correlations of blood DNAm were consistent across CpG sites only in HC participants, with BPD patients showing more variability. This may reflect potential epigenetic dysregulation in BPD, suggesting that blood-derived DNAm may better capture *PRDM8*-related changes in BPD. These findings also highlight the issue of tissue-specificity in epigenetic research, where DNAm patterns can vary substantially across tissues [56], and raise the question of whether peripheral *PRDM8* DNAm changes reflect corresponding patterns in the brain as the primary site of relevance for psychiatric disorders [57]. Based on our results, we cannot conclude any epigenetic correlations between BPD and *PRDM8* DNAm in the brain. However, in psychiatric epigenetics, we are faced with the problem that the tissue of interest—the brain—is not available for molecular analysis in living individuals. We can therefore only report associations in peripheral tissue without providing information about mechanisms in the brain. Furthermore, there are no reports of *PRDM8* DNAm in brain or neuronal tissue available, making it impossible to speculate whether the observed differences in whole blood *PRDM8* DNAm between BPD patients and healthy control individuals could be reflective of the situation in the brain. However, previous reports indicate that *PRDM8* is expressed in neuronal and brain tissue throughout development and in adulthood, indicating a potential role of *PRDM8* in the nervous system [30,31,33,34,35].

Although the increase in BPD patients’ DNAm post-treatment did not remain significant after correction for multiple testing, the direction of the effect is still noteworthy. It aligns with the growing body of research reporting differential DNAm of candidate genes reverting back to HC levels after therapy (for reviews, see, e.g., [58,59]). The hypomethylation of *PRDM8* in the blood of BPD patients at baseline and its nominally significant reversion towards HC levels following therapy further supports the hypothesis of a dynamic epigenetic mechanism. In line with this, we observed a significant inverse correlation between *PRDM8* DNAm levels in blood and BPD symptomatology, suggesting that lower *PRDM8* DNAm may be associated with more severe symptoms. The significant decrease in BSL-23 scores in BPD patients over DBT indicates a meaningful reduction in symptom severity post-treatment, however, without full remission. BPD patients still exhibited significantly higher BSL-23 scores after DBT as compared to the HC groups’ baseline, highlighting the chronic and severe character of BPD. Thus, our results support previous findings of inconsistent reports on BPD remission, recurrence, and diagnosis retention [60,61]. BPD frequently co-occurs with other psychiatric conditions—such as posttraumatic stress disorder (PTSD), major depressive disorder, anxiety disorders, substance abuse, and eating disorders. BPD patients suffering from comorbid diseases frequently display a more pronounced symptom severity and a worse prognosis. In addition, they show poorer response to treatment [5]. However, these comorbidities may not only influence clinical measures but also confound or modulate the epigenetic signatures associated with BPD, including DNA methylation. Unfortunately, information about comorbidities was not available for our sample. However, future studies should include this information to gain more insight into the complex nature of BPD.

*PRDM8* has been reported to play a role in immune regulation, specifically in the transcriptional memory of T cells [36], a pathway increasingly implicated in epigenetic studies of psychiatric disorders (reviewed in, e.g., [62]). Its function as a methyltransferase makes PRDM8 a putative modifier of the histone code, possibly regulating downstream processes relevant for BPD. Specifically, PRDM8 has been shown to reduce transcription of target genes via H3K9 methylation [30,63]. Although intragenic DNA hypomethylation is generally associated with a decrease in gene expression, intragenic *PRDM8* hypermethylation (specifically, cg27242132 and cg19409579) has been reported to correspond to lower *PRDM8* gene expression [64]. Accordingly, a theoretical upregulation of the *PRDM8* gene expression in BPD would potentially correspond to an increase in H3K9 methylation levels. Of note, increased H3K9 trimethylation has been associated with early life stress in animal models [65] and in depressed individuals committing suicide [66]. However, *PRDM8* expression changes have not been consistently linked to its DNAm status [67]. Generally, intragenic DNAm is less extensively characterized than promoter DNAm and may influence not only transcription itself but also alternative splicing regulation [68,69,70]. Further, gene and protein expression and the interplay of complementary epigenetic regulatory mechanisms such as H3K9 trimethylation were not assessed in this study. Future research is warranted to investigate the relationship of *PRDM8* DNAm and its expression, as well as to elucidate potential *PRDM8*-related molecular mechanisms underlying BPD.

No evidence was found in our study that ACE directly impacts *PRDM8* DNAm. Although ACE was strongly associated with BPD diagnosis and symptom severity, it did not correspond to differential *PRDM8* DNAm across tissues. Similarly, no significant correlations were observed between CTQ scores and *PRDM8* DNAm, independent of tissue type. Notably, reclassification of participants based on ACE status led to a shift in group assignments for only 12 participants. Yet, this was sufficient to alter previous significant differences observed in the diagnosis-based group comparisons. This suggests that ACE status may capture biologically relevant variability in *PRDM8* DNAm and highlights its potential importance as a stratification variable in studies investigating the epigenetic landscape of BPD, as has previously been reported for other genes associated with BPD [11].

Our findings suggest that *PRDM8* DNAm is influenced more by BPD and DBT than by ACE, indicating a potential regulation pattern dependent on current psychopathology or treatment context. This partially diverges from previous reports linking *PRDM8* DNAm to early trauma [28,29]. Rivera et al. found hypomethylation at cg18954401 (CpG site 1 in this study) to be associated with prenatal genocide exposure, but only after adjusting for ACE, suggesting an interplay between pre- and postnatal adversity that affects *PRDM8* DNAm [28]. Musanabaganwa et al. identified a different CpG site (cg05059566, located in the 5′ UTR) in genocide-exposed mothers and their offspring, with hypermethylation observed and no reported effect of ACE [29]. These discrepancies may reflect differences in trauma (genocide-related vs. retrospective childhood maltreatment), timing of trauma exposure (pre- vs. postnatal), ethnicity of the sample (Rwandan vs. European), or methodological differences such as the inclusion of BPD patients in our ACE-based analyses, limiting direct comparability. Further, prenatal trauma was not assessed in our cohort, which has previously been implicated as associated with methyltransferase activity, although not *PRDM8* specifically [71]. Thus, while our data do not suggest a direct association of ACE and *PRDM8* DNAm, the broader relationship between pre- and postnatal trauma, *PRDM8* epigenetic regulation, and BPD psychopathology remains to be further elucidated, especially as the lack of prenatal trauma data in our sample hinders the decoding of effects of pre- vs. postnatal trauma on *PRDM8* DNAm. Furthermore, we have to acknowledge that our findings are purely correlational and we cannot establish any causal relationship between *PRDM8* DNAm and BPD or treatment outcome. As we did not perform a longitudinal study, we can also not decipher whether *PRDM8* DNAm might be a consequence of the disorder or whether *PRDM8* DNAm is merely an epiphenomenon coincidental to the pathology or treatment of BPD.

Limitations: The following limitations of our study need to be taken into account when interpreting our findings. First, the unequal distribution of participants with high vs. low levels of ACE across the diagnostic groups hindered the assessment of any interaction effects of these variables. Second, the absence of follow-up data for the HC group restricted the longitudinal analyses to within-subject comparisons in BPD and the comparison to the baseline HC data, making it impossible to account for temporal changes unrelated to DBT. Thus, the lack of follow-up of the HC group warrants cautious interpretation of the observed changes over time. Third, the overall sample size was relatively small, especially the male subsample, which may have obscured effects after controlling for multiple testing. In general, although we included males and females in our study, our study design, while balanced for sex and age between healthy control individuals and BPD patients, was not suitable to decipher sex-specific effects. As DNAm as well as clinical symptom presentation is sex-specific, gender might play an important role and should be investigated further. Furthermore, data on prenatal trauma as well as comorbidities such as PTSD or major depressive disorder were not available, which made disentanglement of pre- and postnatal adversity impossible and may have mediated the association between BPD and DNAm in *PRDM8.* Lastly, gene and protein expression levels were not assessed, which hinders the interpretation of possible functional consequences of the observed DNAm patterns. Broadly, our findings do not enable conclusions about a directionality of effects, i.e., whether *PRDM8* DNAm changes reflect a cause, correlate, or consequence of BPD.

Outlook: Overall, our findings indicate that differential *PRDM8* DNAm is likely to be associated with BPD diagnosis and symptom severity, suggesting it could be a marker of disease state and therapeutic intervention. Building on our insights, while also considering the limitations of our study, future research should be encouraged to explore whether differential *PRDM8* DNAm corresponds to gene and protein expression changes or involves other regulatory mechanisms, such as H3K9 methylation at BPD-related loci. Upcoming studies would profit from larger, well-balanced cohorts including males and females, which enable a stratified analysis by both pre- and postnatal trauma exposure. Such approaches may help to explain the interactions of different trauma types and the epigenetic landscape of BPD. Furthermore, future studies should stratify subjects according to comorbid disorders such as PTSD or major depressive disorder in order to refine our understanding of how comorbidity modulates the epigenetic landscape in BPD and support more personalized therapeutic approaches.

## 5. Conclusions

In summary, our findings indicate that *PRDM8* DNAm is reduced in the whole blood of patients with BPD and shows a nominal increase following DBT, which parallels improvements in symptom severity. While no associations were observed with adverse childhood experiences, the results highlight *PRDM8* DNAm as a potential state-related marker of BPD rather than a direct correlate of early trauma. Importantly, the lack of effects in saliva underscores the relevance of tissue-specific analyses in psychiatric epigenetics. Although preliminary and limited by sample size and the absence of gene and protein expression data, these findings suggest that *PRDM8* may be involved in dynamic epigenetic regulation linked to psychopathology and therapeutic response in BPD. Future research should aim to replicate these results in larger and more diverse cohorts, integrate gene and protein expression measures, and account for comorbidities and trauma timing in order to better elucidate the functional role of *PRDM8* in BPD pathophysiology and its potential as a biomarker of treatment response.

## Figures and Tables

**Table 1:** Demographic and clinical characteristics of the cohort with regard to diagnosis. * = *p*-value of Fisher’s exact test; otherwise, the Wilcoxon rank sum test was used to test for differences between the groups.

Variable	HC	BPD	p (W)
	*n* = 53	*n* = 40	
Age [years] median ± IQR	26.00 ± 9.00	28.00 ± 10.25	0.1266 (1257.00)
Sex female [%]	46 [86.79]	33 [82.50]	0.5751 *
ACE high [%]	10 [18.87]	38 [95.00]	< 0.0001 *
Emotional abuse score median ± IQR	7.00 ± 3.00	17.00 ± 7.25	<0.0001 (1898.00)
Emotional neglect score median ± IQR	8.00 ± 4.00	17.00 ± 8.25	<0.0001 (1830.00)
Physical abuse score median ± IQR	5.00 ± 1.00	9.50 ± 8.50	<0.0001 (1713.50)
Physical neglect score median ± IQR	5.00 ± 2.00	9.50 ± 6.25	<0.0001 (1717.50)
Sexual abuse score median ± IQR	5.00 ± 0.00	10.00 ± 8.50	<0.0001 (1658.50)
CTQ total score median ± IQR	32.00 ± 9.00	59.00 ± 25.25	<0.0001 (1996.50)

## Data Availability

The raw data are available in Table S1 of the Supplement.

## Supplementary Materials

The following supporting information can be downloaded at https://www.mdpi.com/article/10.3390/brainsci15090950/s1: Figure S1: A: No correlation of CTQ score and *PRDM8* blood DNAm pre-therapy was observed (Spearman’s rank correlation test, *p*-value adjusted). B: No correlation of CTQ score and *PRDM8* saliva DNAm was found pre-therapy (Spearman’s rank correlation test, *p*-value adjusted). Table S1: Raw data. Table S2: Spearman correlation analysis results of *PRDM8* DNAm of individual CpG sites with each other, across groups and time points. Table S3: Median BSL-23 scores and DNAm for individual CpG sites and overall *PRDM8* DNAm, according to tissue, group and time point. Sample sizes per variable are additionally displayed. Table S4: Demographic and clinical characteristics of the cohort separated for high and low levels of ACE. * = *p*-value of Fisher’s exact test; otherwise, the Wilcoxon rank sum test was used to test for differences between the groups. Table S5: Median BSL-23 scores and DNAm for individual CpG sites and overall *PRDM8* DNAm, according to separation for high and low levels of ACE. Sample sizes per variable are also displayed.

## Abbreviations

The following abbreviations are used in this manuscript:

BPD	Borderline Personality Disorder
ACE	Adverse Childhood Experience(s)
*PRDM8*	PR domain zinc finger protein 8 (gene)
DBT	Dialectic Behavioral Therapy
DNAm	DNA Methylation
H3K9	Lysine 9 of histone H3
HC	Healthy Control
T1	Pre-therapy
T2	Post-therapy
IPDE	International Personality Disorder Examination
DSM-IV	Diagnostic and Statistical Manual of Mental Disorders, fourth edition
CTQ	Childhood Trauma Questionnaire
BSL-23	Borderline Symptom List
EDTA	Ethylenediaminetetraacetic (tube)
IQR	Interquartile range
