# Supplementary material for: Investigating PRDM8 DNA Methylation in Peripheral Tissues in Borderline Personality Disorder: Association with Symptom Severity but Not Adverse Childhood Experiences
Source: Brain Sci. 2025 Aug 30;15(9):950. doi: 10.3390/brainsci15090950 (PMC12467729; doi:10.3390/brainsci15090950)
Supplement: Supplementary file 1 [file brainsci-15-00950-s001.zip › Supplement_Investigating PRDM8 DNAm in BPD.pdf]

Supplement: Investigating PRDM8 DNA Methylation in Peripheral Tissues in Borderline Personality Disorder: Association with Symptom Severity but not Adverse Childhood Experiences

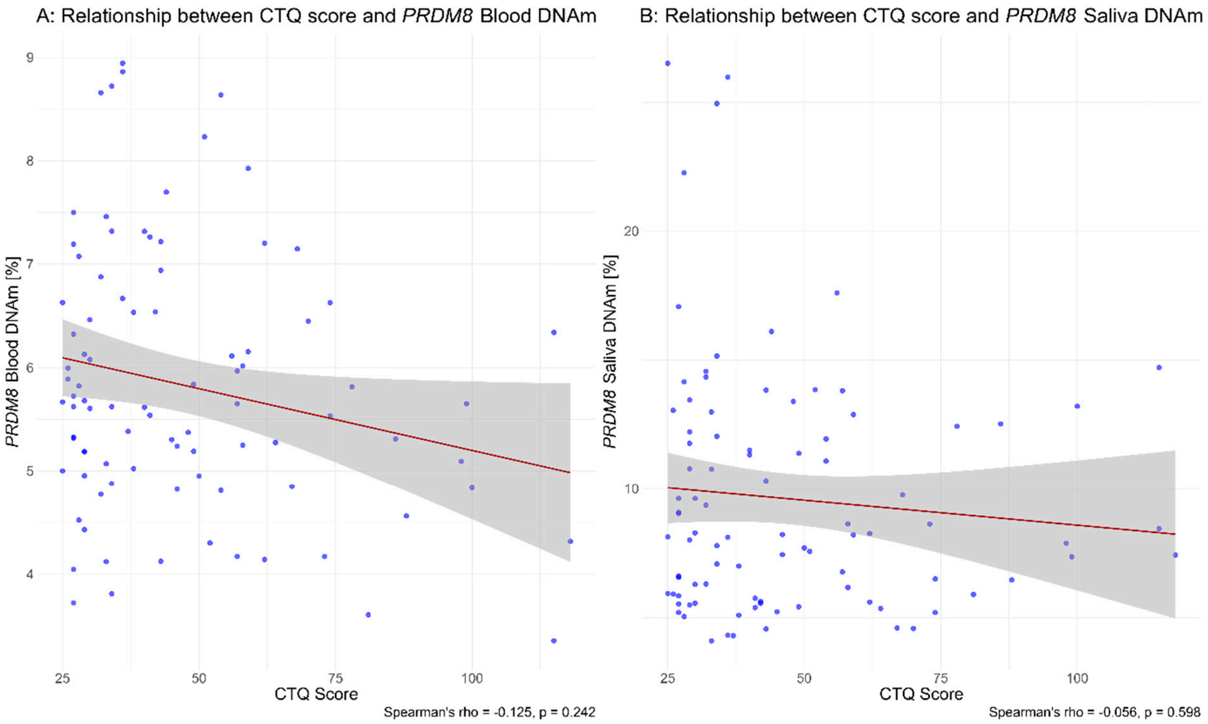

Figure S1: A: No correlation of CTQ score and *PRDM8* blood DNAm pre therapy was observed (Spearman's rank correlation test, p-value adjusted). B: No correlation of CTQ score and *PRDM8* saliva DNAm was found pre therapy (Spearman's rank correlation test).
